# Does Nicotine Prevent Cytokine Storms in COVID-19?

**DOI:** 10.7759/cureus.11220

**Published:** 2020-10-28

**Authors:** Luiz Dratcu, Xavier Boland

**Affiliations:** 1 Psychiatry, South London and Maudsley NHS Foundation Trust, London, GBR

**Keywords:** covid-19, nicotine, cholinergic anti-inflammatory pathway, alpha-7-nach receptor, bipolar affective disorder, chronic obstructive pulmonary disease, cigarette smoking, nicotine replacement therapy, sars-cov-2, pro-inflammatory cytokines

## Abstract

COVID-19 has a benign outcome in most cases, yet it can also be fatal and no specific treatment is available as of yet. Older age and several medical comorbidities are risk factors for COVID-19 complications. We report on an elderly man with a longstanding history of bipolar affective disorder associated with heavy smoking, alcohol abuse and multiple comorbidities, including severe chronic obstructive pulmonary disease and recurrent pulmonary sepsis, who contracted COVID-19 during his inpatient treatment of a manic episode, and who fully recovered from COVID-19 without any need for respiratory support. We discuss how his excessive use of nicotine replacement therapy may have contributed to his emerging unscathed from COVID-19. Nicotine, an α7-nACh receptor agonist, may boost the cholinergic anti-inflammatory pathway and hinder the uncontrolled overproduction of pro-inflammatory cytokines triggered by the SARS-CoV-2 virus, which is understood to be the main pathway to poor outcomes and death in severe COVID-19.

## Introduction

The elderly, the immunosuppressed, and patients with cardiopulmonary disease and diabetes are all at greater risk of COVID-19 complications [1,2]. Chronic obstructive pulmonary disease (COPD) increases 5-fold the risk of severe COVID-19 [3]. Mental illness may also increase the risk of COVID-19 complications [4]. We report on a 63-year-old frail man with the bipolar affective disorder (BAD), severe medical comorbidities, and a history of heavy smoking and alcohol abuse, who contracted COVID-19 during inpatient treatment for a manic episode, and who fully recovered from COVID-19 without any need for respiratory support. We discuss how nicotine replacement therapy (NRT) may have contributed to the favourable outcome of his COVID-19.

## Case presentation

A 63-year-old cachectic white British man, who looked much older than his age, was admitted to our acute psychiatric unit for treatment of a manic relapse of his bipolar affective disorder. He had been previously prescribed oral olanzapine and sodium valproate, but complied erratically with treatment and was admitted following a relapse of his manic symptoms. He was a lifelong smoker (over 100 pack-years), with a history of alcohol misuse (100-120 units/week for years), who suffered from severe chronic obstructive pulmonary disease (COPD) (forced expiratory volume in 1 second 42% of predicted value, forced expiratory volume in 1 second/full vital capacity ratio of 55%, Modified Medical Research Council Dyspnea Score +2) and alcoholic liver disease. In the year leading to the current admission, he had four infective exacerbations of COPD requiring hospitalisation and on one of these admissions required non-invasive ventilation for type 2 respiratory failure. Between exacerbations, his COPD was treated with a regular salbutamol inhaler (a short-acting beta agonist), a regular combination inhaler with umeclidinium (a muscarinic antagonist) and vilanterol (a long-acting beta-agonist), oral hyoscine hydrobromide 300mcg daily (a muscarinic antagonist) and oral carbocysteine 750mg daily (a mucolytic agent). He also had Crohn's disease which required multiple bowel resections, including a right hemicolectomy and a splenectomy. He was on no regular medication for the treatment of his Crohn's disease. In the previous year, a computed tomography colonoscopy revealed a sigmoid lesion suspicious of bowel cancer, but he declined further investigations. He was severely frail (score of 7 on Rockwood Frailty scale) and was completely dependent on others for all aspects of his care. In the first two months, he suffered two episodes of bacterial pneumonia requiring administration of intravenous antibiotics and oxygen, each followed by extended periods of delirium. In between episodes of pneumonia, his presentation fluctuated from periods of confusion to briefer periods of lucidity. His olanzapine was changed for paliperidone long-acting injections. He was offered nicotine replacement therapy (NRT) in nicotine patches (21 mg/day) and inhalators (15mg cartridges, six cartridges/day). He also used e-cigarettes continually (one 3 ml cartridge/day, 18mg/ml) instead of his normal cigarettes. In addition to parenteral nicotine, he inhaled 120-150 mg of nicotine daily after also borrowing e-cigarettes and inhalators from other patients.

**Figure 1:**
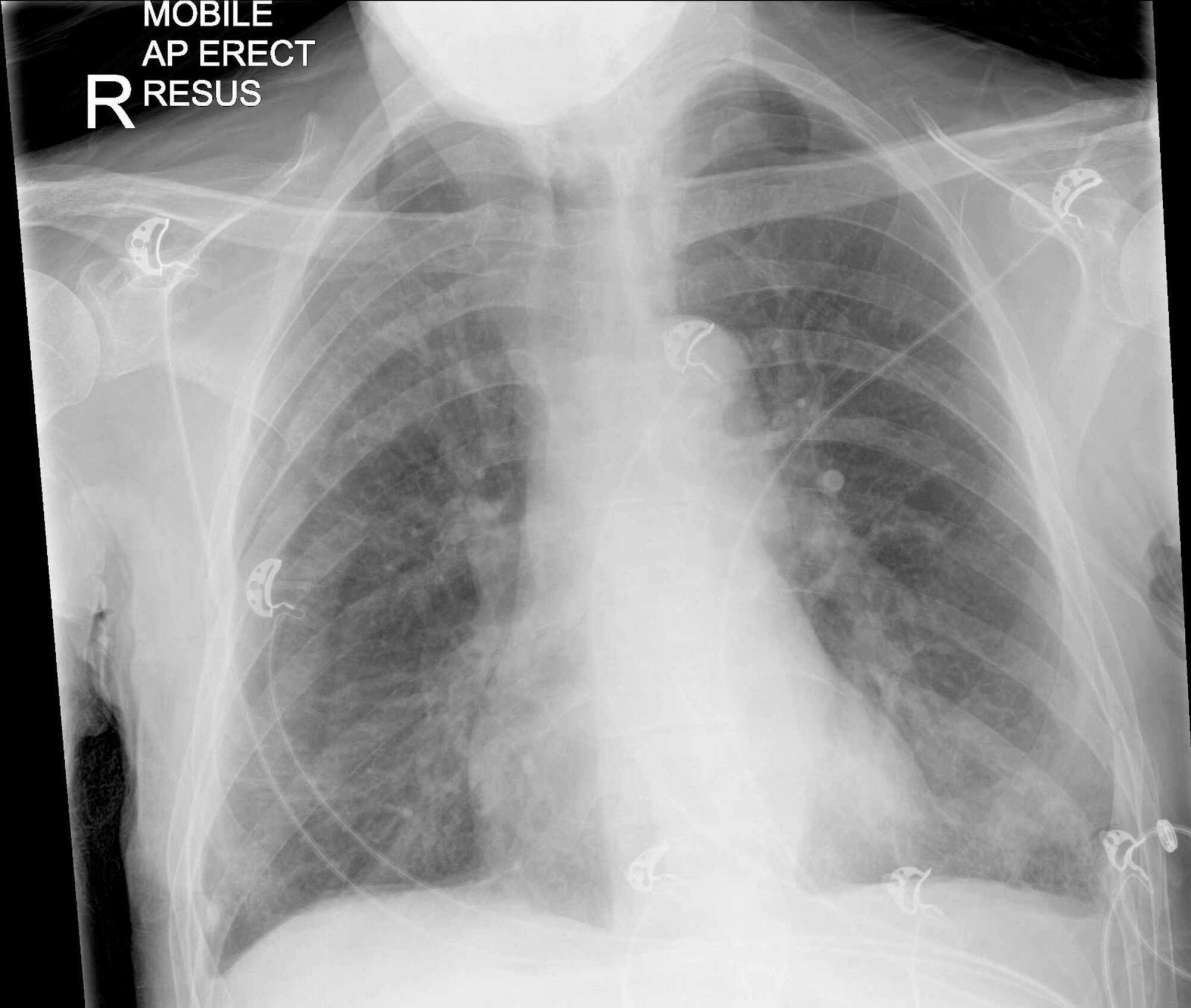
Chest radiograph taken on arrival to accident and emergency Radiograph shows lung hyperinflation, consistent with chronic obstructive airways disease, and diffuse peripheral infiltrates suggestive of viral pneumonia.

Three months into his admission, he became acutely unwell with hypothermia (34.6 Celsius), drowsiness, hypotension (90/70 mmHg), tachypnoea (34 breaths/minute) and hypoxia (oxygen saturation of 79% on air). A nasopharyngeal swab for SARS-COV 2 RNA was positive, and he was transferred to accident and emergency and later a general medical ward. While both C-reactive protein (160 mg/L) and neutrophil count (11.2x109/L) were raised, lymphocyte count was suppressed (1.04x109/L) and other markers of inflammation, including platelet count and alkaline phosphatase levels, were normal. A chest radiograph showed lung hyperinflation consistent with COPD, with bilateral peripheral, infiltrates (figure 1). He received a 7-day course of empirical oral antibiotics, and the hypoxemia was managed with controlled oxygen therapy via a 24% venturi mask only (oxygen flow rate of 1-2 litres/minute). NRT was continued throughout his hospital stay. Three days into this illness, he suffered a self-limiting tonic-clonic seizure. A head computer tomography scan showed no abnormality. Over the following 10 days, he made an impressive recovery, having never required any mechanical ventilator support or anti-inflammatory treatment. He was transferred back to our unit medically well, in good spirits, and with no COVID-19 symptoms.

## Discussion

The course of COVID-19 varies, with up to 80% of patients having no or only mild symptoms, 15% developing pneumonia with or without hypoxaemia, and 5% progressing to acute respiratory distress syndrome (ARDS) and multiorgan involvement [1]. Some patients progress suddenly from mild dyspnoea to ARDS requiring urgent ventilatory support. The median time from first symptoms to hospitalisation and ARDS is eight days [2]. However, ten days after exhibiting COVID-19 symptoms, our patient never experienced any complication other than a seizure unrelated to COVID-19 [5]. 

His unscathed recovery defied our worst predictions. He might just have been lucky. COVID-19 is a novel disease that is not yet fully understood, and most of those who fall ill suffer flu-like symptoms lasting a week or so. He was, however, an older man with a bipolar affective disorder associated with alcohol abuse. He was a heavy smoker and suffered from COPD, inflammatory bowel disease, recurrent chest sepsis and probable bowel cancer. He also was asplenic. As a patient with significant risk factors for COVID-19 complications, another plausible explanation is that he may have been spared from the "cytokine storms" that can be triggered by SARS-CoV-2 virus infection. 

Inflammation is the body's first line of defence against infection, and microbes have evolved strategies to avoid precipitating inflammatory responses. However, some pathogens, like the influenza virus and the Gram-negative bacterium Francisella tularensis, do trigger life-threatening "cytokine storms" in the host [6]. "Cytokine storms" also seem to contribute to severe COVID-19 [7]. In the lungs, the angiotensin-converting enzyme two receptors (ACE2), the principal receptor for the SARS-CoV-2 virus, is highly expressed on epithelial cells, through which the virus enters the organism [8]. In the absence of neutralising antibodies, the ensuing cellular and cytokine inflammatory response in the infected lungs is capable of clearing the virus but can cause severe impairment of lung function [7]. Damage to alveolar pneumocytes and release of inflammatory mediators, along with activation of neighbouring dendritic cells, attract macrophages, then T-lymphocytes, and cause the release of pro-inflammatory interleukins and TNF-α. The overproduction of pro-inflammatory cytokines, together with the activation of the coagulation cascade and microthrombi formation in the lung vasculature, may lead to ARDS and later to multiorgan failure and death [7]. 

The multiple severe diseases from which our patient suffered might have compromised his immune system, thereby paradoxically protecting him by reducing the likelihood of an uncontrolled inflammatory response to the virus. Crohn's disease, which, in his case necessitated several surgical interventions, has been associated with immunodeficiency of macrophages [9]. Bowel cancer may likewise hinder an excessive inflammatory response. The spleen is involved in cytokine production following infections, but patients who have had a splenectomy, as this man has, are at increased risk of bacterial sepsis; in the UK, people with splenectomy are included in the shielding list for COVID-19. Moreover, he received antipsychotics, and there is evidence that antipsychotics may reduce inflammatory activity [10]. However, he also had alcoholic liver disease, which in turn may activate innate liver immunity and the expression of pro-inflammatory cytokines [11]. 

Whether the combined effect of his medical comorbidities prevented a "cytokine storm" is, however, a matter for speculation. What is clear is that his immune system was healthy enough to enable him to fully recover from COVID-19 unaided by any treatment, except for empirical antibiotics and, perhaps, nicotine. His heavy smoking, the likeliest cause of his severe COPD and an exacerbator of his Crohn's disease, was also a source of large amounts of nicotine to which he was addicted. Throughout his admission, he used NRT in copious amounts. His craving for hefty doses was obvious when his cigarettes were replaced by NRT, which he supplemented with nicotine from e-cigarettes, reaching an estimated consumption equivalent to over 5-6 packs of cigarettes a day. In the light of emerging evidence of a possible role of nicotine on the clinical course of COVID-19, and also of our patient's pre-existing poor medical state, his continuous use of such excessive amounts of nicotine would be hard to ignore, as it may not only account for his low body temperature at the onset of his COVID-19 symptoms but may also have played a decisive role in the outcome of his illness. 

Given the association of smoking with COPD, smokers would be expected to be particularly vulnerable to COVID-19 complications [3,12]. However, a retrospective cohort study in France reported that smokers had a SARS-CoV-2 infection attack rate four times lower than non-smokers [13]. Another retrospective French study reported that, compared to the general population, smokers had a dramatically lower risk of developing symptomatic or severe COVID-19 [14]. Further similar findings elsewhere [15,16] have raised the question as to whether nicotine may have any biological effect on the SAR-CoV-2 virus. 

Nicotine can selectively reduce the inflammatory response in various infection states, including Legionella pneumophila and Chlamydia pneumoniae infection, via the cholinergic anti-inflammatory pathway [6]. Nicotine is an agonist at the α7 subunit of nicotinic acetylcholine (α7-nACh) receptors on innate immune cells such as macrophages. These receptors respond to acetylcholine from different sources, including other immune cells and the vagus nerve, and their activation causes suppression of pro-inflammatory cytokines. Nicotine is able to suppress the production of pro-inflammatory cytokines by mimicking the binding of acetylcholine. 

The SARS-CoV-2 virus may itself antagonise the nACh receptor pathway and reduce its anti-inflammatory action [17]. Nicotine, again through its action at α7-nACh receptors in the lungs, could prevent the virus-induced nACh receptor dysregulation by activating the cholinergic anti-inflammatory pathway. Smoking could thus attenuate the normal defensive function of the immune system and reduce the hyperinflammatory response seen in severe COVID-19, while the immune system of non-smokers may be more prone to SARS- CoV-2 cytokine release syndrome [17]. However, as nicotine increases the expression of ACE2 in the lung and ACE2 increase is mediated by α7-nACh receptors, smoking may promote cellular uptake mechanisms of SARS2 CoV-2 through α7-nAch receptor signalling [18]. 

Current evidence for a protective effect of nicotine in COVID-19 remains controversial. Nonetheless, there has been a support to the notion of repurposing NRT products [19], such as nicotine patches [20], as an adjunctive treatment for COVID-19 in smokers as our case seems to suggest, the potential role of NRT in the management of COVID-19 warrants further scrutiny.

## Conclusions

In the absence of any effective treatment for COVID-19, further research as to whether nicotine replacement offers protection against severe SAR-CoV-2 infection in smokers is clearly essential. If the mechanisms through which nicotine may interact with the virus remain speculative, the effects of route of administration, duration, dosing and frequency of use of nicotine on any such interaction are unknown. Should NRT be found to be of help in the management of COVID-19, it would be yet another strong reason to persuade smokers to switch to NRT and ultimately quit smoking.
